# Use of Smartphone-Based Experimental Data for the Calibration of Biodynamic Spring-Mass-Damper (SMD) Pedestrian Models

**DOI:** 10.3390/s25051387

**Published:** 2025-02-24

**Authors:** Chiara Bedon, Martina Sciomenta, Alessandro Mazelli

**Affiliations:** 1 Department of Engineering and Architecture, University of Trieste, 34127 Trieste, Italy; 2 Department of Civil, Construction-Architectural and Environmental Engineering, University of L’Aquila, Monteluco di Roio, 67100 L’Aquila, Italy

**Keywords:** Human-Structure Interaction (HSI), Spring-Mass-Damper (SMD) model, timber floors, experiments, smartphone-based acquisitions

## Abstract

In practice, the structural analysis and design of pedestrian systems subjected to human-induced vibrations is often based on simplified biodynamic models that can be used in place of even more complex computational strategies to describe Human-Structure Interaction (HSI) phenomena. Among various walking features, the vertical reaction force that a pedestrian transfers to the supporting structure during motion is a key input for design, but results from the combination of multiple influencing parameters and dynamic interactions. Robust and practical strategies to support a realistic HSI description and analysis have hence been the object of several studies. Following earlier research efforts, this paper focuses on the optimised calibration of the input parameters for the consolidated Spring-Mass-Damper (SMD) biodynamic model, which reduces a single pedestrian to an equivalent SDOF (with body mass *m*, spring stiffness *k*, and viscous damping coefficient *c*) and is often used for vibration serviceability purposes. In the present study, this calibration process is carried out with smartphone-based acquisitions and experimental records from the Centre of Mass (CoM) of each pedestrian to possibly replace more complex laboratory configurations and devices. To verify the potential and accuracy of such a smartphone-based approach, different pedestrians/volunteers and substructures (i.e., a rigid concrete slab or a timber floor prototype) are taken into account, and a total of 145 original gaits are post-processed for SMD modelling purposes. The analysis of the experimental results shows a rather close match with previous findings in terms of key pedestrian parameters. This outcome poses the basis for a more generalised application of the smartphone-based strategy to a multitude of similar applications and configurations of practical interest. The validity of calibration output and its possible sensitivity are further assessed in terms of expected effects on substructures, with a critical discussion of the most important results.

## 1. Introduction

The continuous innovation in architectural and structural concepts and materials for constructions and building components makes increasing the number of possible engineering applications that are based on the design of slender and/or lightweight pedestrian systems important [1]. These pedestrian solutions, both for the materials and technical details in use, can possibly be subjected to high sensitivity towards human traffic and human-induced vibrations, and special care is commonly required for those systems where the fundamental vibration frequency is lower than 8 Hz [2]. As such, dedicated calculation methods are typically required for analysis and verification [3,4].

**Figure 1:**
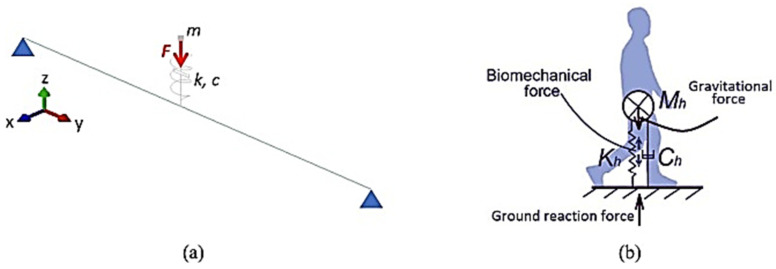
Biodynamic pedestrian modelling: (**a**) example of a simple structural model for HSI analysis and (**b**) schematic representation of Spring-Mass-Damper (SMD) pedestrian. Figure reproduced from [10] with permission from © Elsevier, under the terms and conditions of a Creative Commons CC-BY 4.0 license agreement.

On the side of pedestrian-induced effects, simplified and empirical methods can be used, or even more robust modelling and simulation strategies, to account for the dynamic response of the structure (Figure 1), as well as for the correlated Human-Structure Interaction (HSI) phenomena [3]. Among others, Spring-Mass-Damper (SMD) biodynamic models have been widely investigated in the literature to reproduce the effect of single pedestrians [5,6,7,8]. Furthermore, they still present some challenges and uncertainties for the calibration of input parameters and possible sensitivity to the structural background and its dynamic features compared to pedestrians. Indeed, the use of general SMD formulations can be particularly helpful and efficient for those pedestrian systems that, like timber structures, while moderately sensitive to human-induced vibrations, could require specific calculations [1,4,9].

To this aim, an experimental and numerical study was originally proposed in [10] for an optimised SMD model characterisation based on tracked records from a single body sensor fixed in the Centre of Mass (CoM) of pedestrians (Figure 1b). The effect of pedestrians moving on transparent floors made of glass was also investigated in [11], highlighting some important dynamic effects due to specific mass and vibration frequency ranges. The Dynamic Load Factor (DLF) was also calculated and compared to studies in the literature for different transparent or opaque substructures, with vibration frequencies down to 7.28 Hz and structure-to-pedestrian mass ratios in the order of ≈5.75.

Following earlier experiences, the present study further explores the SMD calibration based on single-body sensor measurements, according to [10], and adds some important advancements for its optimisation. In particular, this paper verifies the possible validity of smartphone-based acquisitions to track CoM motion during walks and uses them as a key input for the SMD calibration. Various researchers have addressed the potential of smartphone devices for structural health monitoring purposes [12,13,14] and to track the dynamic response of several structures.

Notably, the present study verifies the use of smartphone-based acquisitions from the perspective of pedestrians and within the framework of uncoupled body measurements for a robust mathematical description of human-induced reaction forces on substructures [10,15]. This last option represents a major optimisation of the calibration strategy, assuring that smartphone devices can be used in place of more sophisticated sensors. Moreover, the additional goal of the present study is represented by the possible extension of the validity of the SMD calibration strategy presented in [10] pertaining to different configurations of technical interest. For the original experimental analysis, two volunteers are involved in the dynamic tests. Moreover, in addition to using a rigid concrete slab for the walks, a full-scale timber floor prototype is used for the robust extension of the technical configurations that are taken into account in the experimental program.

## 2. Investigation Strategy

### 2.1. Past Smartphone-Based Ivestigations for Pedestrian Behaviours

The use of smartphones for diagnostic purposes increasingly represents, for many research fields, a low-cost, easily accessible and efficient tool in support of rapid but sufficiently realistic investigations. Notably, the specific implementation of smartphones in the analysis of pedestrian behaviours and motion features belongs to one of the so-called sensor-based strategies but represents only a single task of a rather wide and challenging topic, which has attracted the interest and need for practical tools from many different disciplines [16]. Sensor-based approaches for the recognition and analysis of gaits are a possible alternative to other available techniques, which include video image processing approaches or radio-based methods [16,17,18].

Peng et al. [19] used smartphone-driven videos to track the walking and running features of a group of pedestrians and mathematically derived some important quantitative features of their motion, including the ground reaction force. Min et al. [20] also investigated the kinematic and kinetic gait features of pedestrians. They took a major advantage from a smartphone-based motion capture system able to track videos of moving pedestrians and explored some important aspects of their walking features (i.e., pelvic tilt, hip flexion, knee extension, and ankle dorsiflexion), with specific attention paid to patients with neurological disorders.

Contreras et al. [21] verified the accuracy of smartphone-based measurements for older and diseased pedestrians by comparing the obtained walking parameters with highly refined motion-capture laboratory tracking records. The focus of their research was the analysis of acceleration and angular data registered simultaneously by two different smartphones, fixed to the legs of each pedestrian, and set to a sampling rate of 100 Hz. The analysis of comparative results reported in [21] confirmed the high accuracy of records from the built-in sensors of common smartphones and their possible efficient use for medical applications.

Suzuki et al. [22] proposed a smartphone-based application to measure human-induced reaction times during walks. Their experimental study was based on the comparative use of two different smartphone devices (Nexus 6 (Motorola Mobility LLC., Libertyville, IL, USA) and Galaxy S II WiMAX (Samsung Electronics Co., Seoul, Republic of Korea)) to investigate the reaction time and quantify the possible risk of falling for pedestrians when walking and talking together. It has been demonstrated by several studies that the use of smartphones while walking can modify the motion features, and these effects can be quantified in a typical increase in the step width and knee abduction moment [23].

The need for efficient investigation tools and low-cost wearable sensors for gait analysis in many different applications (i.e., medical and others) has also been demonstrated by other research studies; see, for example, refs. [24,25,26,27,28].

### 2.2. Present Study: Goal, Impact, and Limitations

The present investigation takes advantage of a single smartphone device to track (from its built-in sensor) some important quantitative features of body motion and elaborates on them to derive a sound calibration of input properties for the SMD pedestrian model in Figure 1. Most importantly, following [10], attention is given to acceleration and inclination records in the time interval of normal walks, which are post-processed to calculate the basic parameters of the SMD model. In this sense, from a technological point of view, the study is in line with some recent literature applications in which wearable inertial sensors were used to quantify the human-induced effects of pedestrians when walking or running [29,30,31] and demonstrated the high accuracy, efficiency, and potential of similar acquisitions.

In terms of SMD calibration, it is important to remember that the so-collected smartphone-based records are presently elaborated for a selection of adult pedestrians in good health condition and walking normally on different substructures. The primary goal is to obtain the key input parameters for the SMD calibration process (see Section 2.3) and verify the lack of major issues in the overall procedure, as far as commercially built-in smartphone sensors are used in place of more sophisticated instruments. The final application of such a smartphone-based validated model—and in particular the corresponding description of human-induced effects on pedestrian structures—is expected to take form in the vibration serviceability analysis of new or existing floors, as well as in a possible rapid diagnostic investigation for in-service pedestrian systems.

To this aim, it is important to recall that the consolidated SMD model object of study has been investigated by many researchers pertaining to the description of pedestrians on various substructures [3,5,6,8]. Furthermore, in most cases, rather complex technologies have been used (i.e., requiring multiple sensors and laboratory instruments). Accordingly, instances in the literature have presented different calibration strategies and procedures for the derivation of the basic SMD parameters of a given pedestrian, with possible consequences for structural effects [32]. This is not the case for the present application, in which the possible generalised use of a portable, commercial smartphone is assessed.

### 2.3. Reference Theoretical Model for SMD Pedestrian Model Calibration

The basic assumption of the present strategy aligns with the study reported in [10], in which it is assumed that the vertical force *F_z_*(*t*) in time *t*, induced by a pedestrian with mass *M*, is proportional to the CoM acceleration *a_z_*(*t*) and can be estimated from Newton’s second law of motion:

(1) $$F_{Z}\left(t\right)=Ma_{Z}(t)$$

In practical terms, Equation (1) represents the reaction force that a pedestrian induces at each step on the substructure. According to [10], however, it is also important to remember that *F_z_*(*t*) is implicitly proportional to two additional critical parameters, namely:

- the SDOF stiffness *k* (which is representative of pedestrian’s legs);
- and the vertical motion path of body CoM, Δ*h*(*t*), which further depends on the CoM trajectory and can be possibly affected by the floor flexibility:

(2) $$F_{Z}\left(t\right)=k\cdot ∆h(t)$$

By totalling Equations (1) and (2), the basic SMD properties (*k*, *c*) can thus be efficiently calculated. It is important to note, in this context, that the experimental calibration proposed in [10] was based on multiple walks from a single pedestrian. In addition, the reference mathematical model considers that the lower the floor rigidity, the lower the measured leg stiffness, and thus the corresponding CoM acceleration and vertical trajectory modification. As such, validation is required for different scenarios.

Regarding damping, assuming that *m* = *M*, the calibration approach is based on the definition of undamped and damped frequencies for the SDOF pedestrian:

(3) $$f_{m}=\sqrt{\frac{k}{m}}\cdot \frac{1}{2\pi }$$

(4) $$f_{md}=f_{m}\cdot \sqrt{1-\xi^{2}}$$

The estimation of the damping ratio *ξ* and corresponding viscous damping coefficient *c* are in fact obtained from iterative calculations in terms of Equations (3) and (4), considering that:

(5) $$\xi =\frac{c}{2m\omega_{m}}$$

and

(6) $$\omega_{md}=\omega_{m}-\sqrt{1-2\xi^{2}}$$

where *w_m_* and *w_md_* represent the associated undamped and damped pulsations.

**Figure 2:**
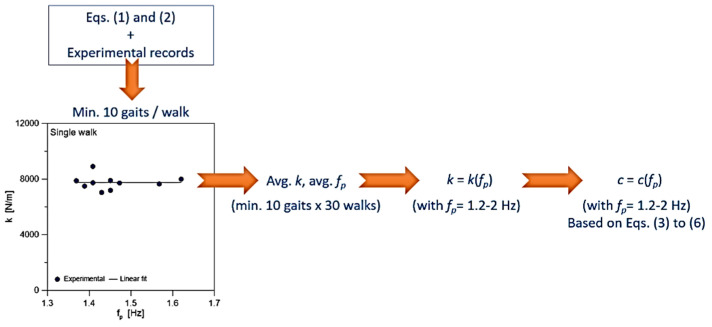
Flowchart for the experimental derivation of spring stiffness *k* and damping coefficient *c* parameters for the SMD biodynamic model presented in [10]. Figure reproduced with permission from © Elsevier, under the terms and conditions of a Creative Commons CC-BY 4.0 license agreement.

The iteration in Equations (3)–(6) must be repeated in terms of the viscous damping coefficient *c*, until the estimated frequency converges (Figure 2).

In practical terms, an efficient SMD calibration was finally proposed in [10] as a function of the pacing frequency *f_p_* (in Hz, with 1.2–2 Hz the explored range):

(7) $$k=8190f_{p}-4315.8\;\;\;\;\;(R^{2}=0.91)\;\;\;\mathrm{in}\;N/m$$

(8) $$\xi =0.5915f_{p}-0.3375\;\;\;\;\;(R^{2}=0.52)$$

(9) $$\xi =1.0705-0.0002c\;\;\;\;\;(R^{2}=0.84)$$

with *m* = *M*.

## 3. Dynamic Experimental Analysis

For the purpose of the present investigation, an extended experimental study was carried out in Italy in joint collaboration between research members from the University of Trieste and the University of L’Aquila. The latter provided major practical support in the arrangement of the bespoke laboratory setup. The dynamic experiments herein reported took place in August and November 2024.

### 3.1. Full-Scale Timber Floor Prototype

To perform the non-destructive dynamic tests, a full-scale timber floor was built at the Laboratory of Materials and Structures of the University of L’Aquila, Department of Civil, Construction-Architectural, and Environmental Engineering.

**Figure 3:**
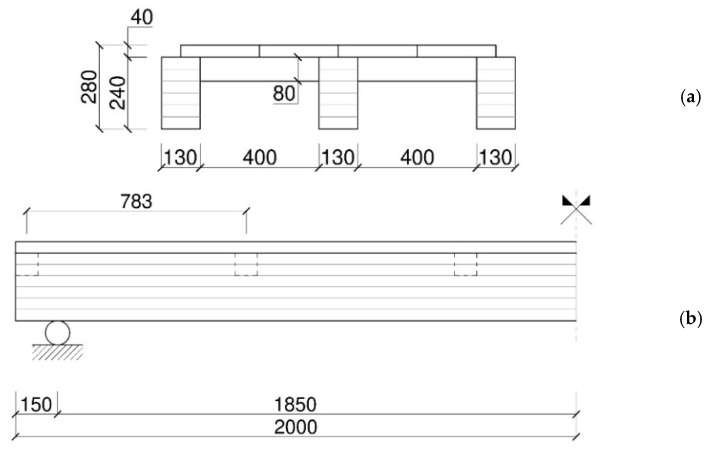
Timber floor prototype for non-destructive dynamic tests: (**a**) cross-section and (**b**) lateral view (nominal dimensions in mm).

According to Figure 3, the specimen was assembled to have a nominal span *L* = 4000 mm and a distance between supports of 3700 mm. The resisting cross-section of the floor consisted of three glulam longitudinal beams (130 × 240 mm, their section), spaced 530 mm apart (Figure 3a). The basic mechanical properties of the glulam material were obtained through previous four-point bending tests, as discussed in [33], and carried out according to EN 408 standard [34]. The tests resulted in mean and characteristic values of *f_m,k_* = 40 MPa, *E_0,mean_* = 14,360 MPa, and *ρ_m_* = 695 kg/m^3^.

A single solid wood plank layer, 40 mm in thickness, was placed on the top of the beams to create the pedestrian surface. A set of 12 boards (arranged 4 in width × 3 in span), with a width of 265 mm and a length of 1600 mm (800 mm at mid-span), was used to cover the floor surface. The plank layer was made of D24 timber, with nominal properties of *f_m,k_* = 24 MPa, *E*_0*,mean*_ = 10,000 MPa, and *ρ_m_* = 580 kg/m^3^ [35,36]. The mechanical properties of solid wood (D24 type) were quantified by visual classification. In addition, secondary transversal beams made of solid wood (D24 resistant class), with an 80 × 80 mm cross-section and a total length of 400 mm, were placed 783 mm apart from each other orthogonally to the longitudinal beams to provide a lateral restrain and support for the plank. Finally, the mechanical connection between the plank layer and the longitudinal glulam beams was obtained using 45° inclined screws (9 mm in diameter and 160 mm in length), spaced 200 mm apart. The serviceability stiffness of such a kind of connection—which has a primary effect on the out-of-plane bending stiffness and response of the floor—was evaluated by means of previous destructive push-out tests carried out at the same laboratory, according to the EN 26891 standard [37], as discussed in [33]. The experiments resulted in an average stiffness of *K_ser_* = 5.97 kN/mm for a single fastener. Accordingly, the maximum resistance for a single fastener was predicted to be ≈20 kN [33].

**Figure 4:**
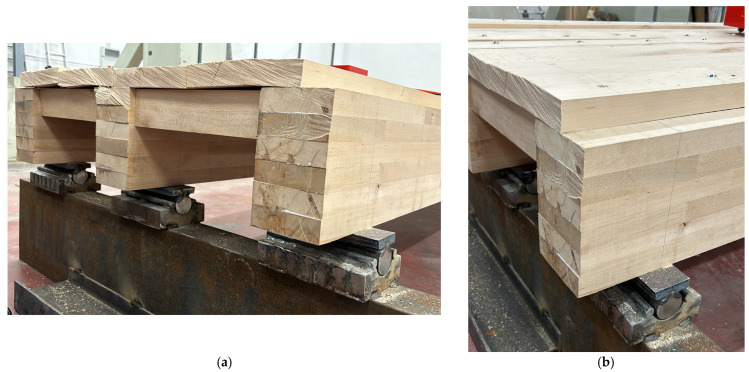
Detail of (**a**) left and (**b**) right end supports for the timber floor prototype.

Regarding the mechanical boundaries, simply supported restraints were reproduced with bespoke steel rollers, placed 180 mm apart from the ends (Figure 4).

### 3.2. Structure-to-Pedestrian Parameters

Many influencing parameters should be commonly taken into account when exploring the human-structure interaction and the associated vibration issues. For the present study, the total weight of the floor prototype was estimated at ≈370 kg, thus resulting—for the scheduled vibration tests—in a structure-to-pedestrian mass ratio in the order of ≈ 5 (with ≈70 kg the weight of volunteers).

**Figure 5:**
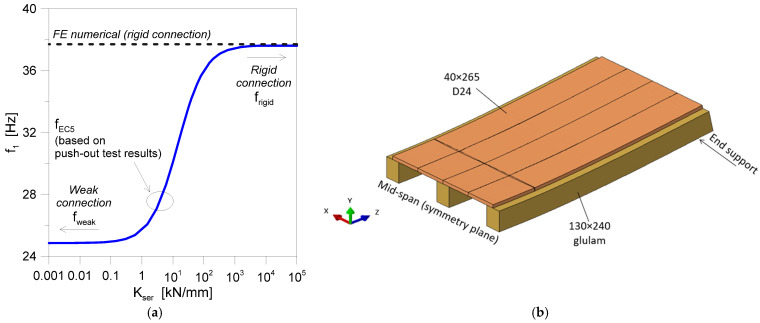
Dynamic estimates on the empty floor prototype: (**a**) analytical fundamental vibration frequency, according to Eurocode 5, as a function of the connection stiffness, and (**b**) an example of a beam-like fundamental deformed shape (for half the nominal geometry) in the presence of a rigid connection.

Another critical parameter is represented by the fundamental vibration frequency of the floor and possible resonance issues with pedestrians. Based on preliminary analytical estimates that were carried out according to the Eurocode 5 for the empty floor, its beam-like fundamental vibration frequency in a simply supported condition was predicted at about *f*_EC5_ ≈ 28.5 Hz, which means a still rigid (but relatively light) pedestrian structure compared to normal walking features. It is important to remember, however, that the serviceability stiffness of the connections in use is a major influencing parameter on the structural side [1]. In this sense, a typical trend of analytical fundamental vibration frequencies for the empty floor prototype is shown in Figure 5a as a function of the connection stiffness. Whilst the global performance of the floor can still be associated with a beam-like response (Figure 5b), the connection in use was expected to provide a rather weak bond compared to a fully rigid connection. The calculated *f*_EC5_ value resulted in a relatively small rigid configuration (*f*_rigid_ = 37.6 Hz) and was rather close to a weak connection (*f*_weak_ = 24.8 Hz).

### 3.3. Instruments

**Figure 6:**
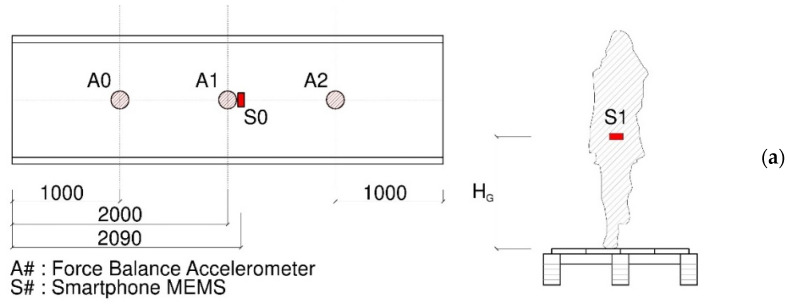

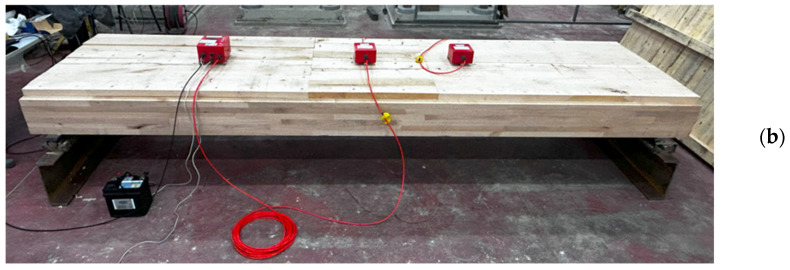
Dynamic tests: (**a**) schematic representation of instrumental setup (dimensions in mm) and (**b**) general view of the floor prototype before testing.

The experimental program was carried out according to the test setup schematised in Figure 6a. To perform the dynamic tests, the floor was instrumented with three force balance accelerometers, 1.5 kg in weight each, placed in the A0 and A2 positions (¼*L* and ¾*L*) and in A1 (the mid-span section). The sample rate of these accelerometers was set at 200 Hz. Furthermore, a smartphone (S0 sensor) was fixed to the floor for the whole experimental campaign, close to the central accelerometer A1, in order to collect the vertical acceleration data with a sample frequency of 250 Hz. The S0 sensor consisted of a Xiaomi Redmi Note 7 device with 4 GB RAM and an octa-core processor (2.20 GHz maximum). Finally, for the SMD model calibration, a dedicated smartphone was fixed to the body CoM of each pedestrian (the S1 sensor in Figure 6a) and secured by the belt in the H_G_ position.

In this regard, it is important to remember that the primary goal of the present investigation is a validation of the calibration strategy for an SMD pedestrian, like in Section 2, and in particular its application to different substructures compared to [10], by using smartphone-based body CoM measurements for the experiments (S1 sensor).

**Figure 7:**
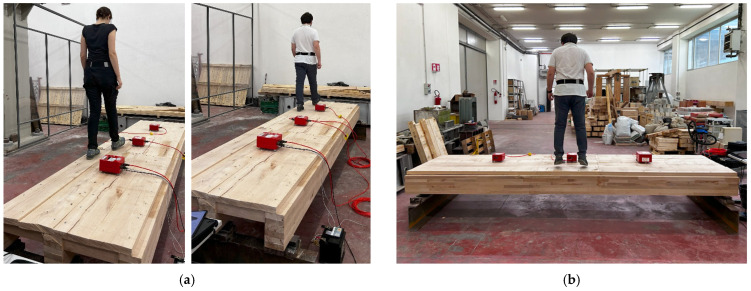
Dynamic tests: examples of (**a**) normal walking configurations and (**b**) jumps.

The achievement of such a goal took advantage of a more extended experimental program carried out at the University of L’Aquila, from Summer 2024, which involved three different pedestrians in total, a multitude of walking configurations, and a specific focus on structural vibration issues. The dynamic test configurations included a combination of single or double pedestrians and even jumps (Figure 7). The complete test schedule achieved a total of 33 possible loading combinations for the timber floor prototype, with variations in the walking features (*f_p_* = 1–2 Hz, the investigated frequency range) in the walking path of the involved pedestrians (i.e., linear or random). For the present SMD application, a selection of experimental records (i.e., a single pedestrian crossing linearly on the floor) was taken into account (Section 3.4).

### 3.4. Examined Test Configurations

Two adult pedestrians were involved in the experimental analysis (p_2_ and p_3_ in Table 1). For comparative purposes, Table 1 summarises the reference parameters for the original study carried out in [10], considering multiple random walks (with 300 gaits in total) for a single pedestrian (p_1_) on a rigid concrete using the laboratory foundation system. Notably, a Wi-Fi MEMS triaxial accelerometer was used in [10] in place of a commercial smartphone with a sampling rate of 200 Hz. In this regard, to facilitate the comparison of present and past experimental evidence, the p_2_ volunteer was asked to track the body CoM records both when walking on the timber floor prototype and on a rigid concrete foundation slab (next to the timber floor in the laboratory). The same smartphone (S1 sensor) was used for the p_2_ and p_3_ volunteers. Its sampling rate was set at 100 Hz, which is common to most commercially available smartphone devices.

In terms of experimental acquisitions, due to the floor dimensions, each walk on the timber prototype consisted of approximately seven gaits, depending on the walking speed (Table 1). For comparative purposes, the same number of gaits and stride lengths was taken into account for p_2_ when walking on the rigid concrete slab.

## 4. Analysis of Experimental Results

### 4.1. Experimental Observations and Post-Processing

**Figure 8:**
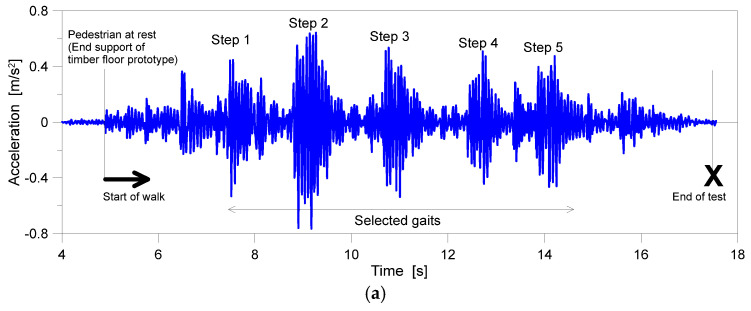

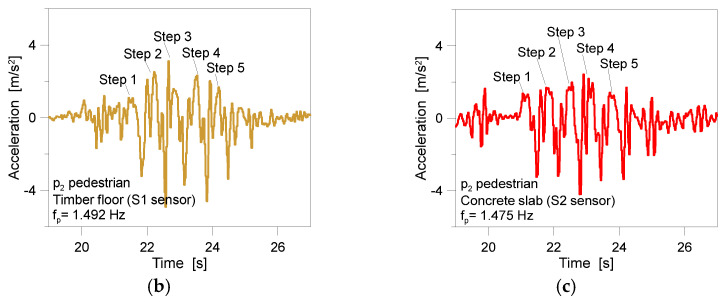
Example of test results: acceleration records (**a**) at the mid-span section of the timber floor and (**b**,**c**) detail from the body CoM acquisitions of pedestrian p_2_ (S1 sensor) when walking on the timber floor or rigid concrete slab, respectively.

Generally, the experimental results were found to agree with Figure 8 in terms of acceleration at the mid-span section of the timber floor (Figure 8a) or the body CoM acceleration for pedestrian p_2_ when walking, respectively, on the timber floor (Figure 8b) or on the rigid concrete slab (Figure 8c).

For post-processing, as shown in Table 1 and [10], the first and last steps of each registration, as shown in Figure 8b,c, were disregarded, and the derivation of key SMD parameters was based on the central gaits for each experimental walk. The motion parameters for the first and last steps in a given walk were commonly characterised by limited amplitude and speed, compared to the others, as a direct consequence of walk patterns starting from rest and stopping at the end of the floor.

**Figure 9:**
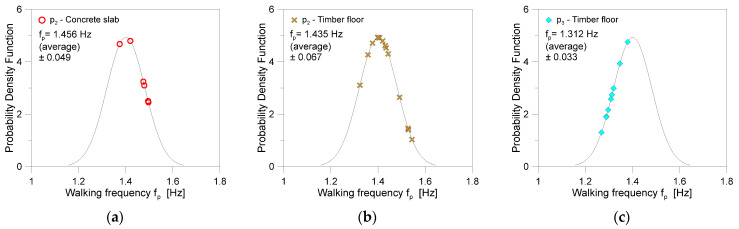
Gaussian distribution of average walking frequencies *f_p_* for the post-processed experimental walks of (**a**,**b**) p_2_ and (**c**) p_3_ pedestrians.

Moreover, the volunteers were required to move linearly during the individual registrations and, as much as possible, fix the walking frequency *f_p_* for each scenario. Furthermore, the use of additional devices (i.e., metronomes) was avoided to impose a specific rhythm. This means that each walk was characterised by some deviations of motion frequency compared to the mean value; see Table 2 and Figure 9.

All the relevant motion parameters for the SMD model were hence calculated as a function of the average *f_p_* of each walk, with:

(10) $$f_{p}=avg(f_{gait,1},\ldots f_{gait,5})$$

In total, the average walking frequency from 29 walks (and 145 gaits for p_2_ + p_3_ configurations) was measured at 1.402 Hz (±0.082).

**Figure 10:**
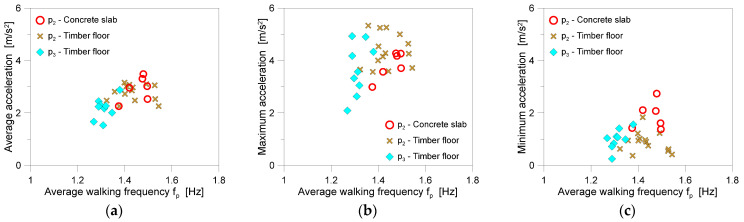
Measured vertical body acceleration for each experimental walk as a function of the walking frequency *f_p_* in terms of (**a**) average, (**b**) maximum, and (**c**) minimum values.

As a basic input parameter, according to Equations (1) and (2), the vertical body acceleration was used as a primary experimental input for the derivation of SMD features. The variability of motion features during the experiments was accounted for in terms of average acceleration for each walk (Figure 10a). For comparative purposes, Figure 10 also shows a comparison of mean acceleration trends, with the corresponding maximum and minimum peaks in each walk, as a function of *f_p_* (Equation (10)).

### 4.2. Smartphone-Based Derivation of SMD Biodynamic Parameters

The derivation of stiffness and damping parameters for the SMD model was based on sets of experimental records grouped by pedestrian (p_2_ or p_3_) and substructure (timber floor or concrete slab).

**Figure 11:**
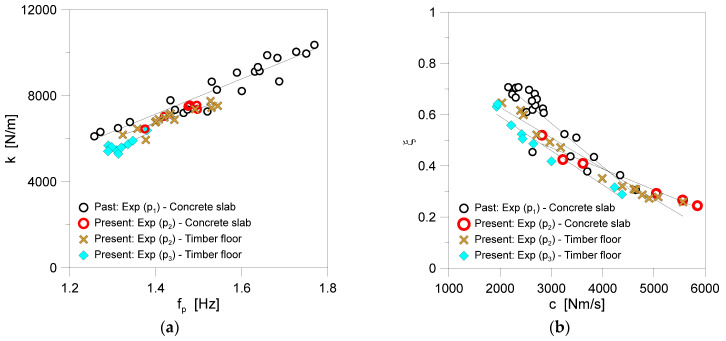
Experimental derivation of biodynamic model parameters for pedestrians p_2_ and p_3_ (present study) compared to earlier findings for pedestrian p_1_: (**a**) spring stiffness *k*, as a function of walking frequency *f_p_*, and (**b**) damping ratio *ξ*, as a function of damping coefficient *c*.

The typical results can be found in Figure 11, where each dot at a given frequency *f_p_* represents the output of the average calibration for a single walk. Overall, it can be seen from Figure 11 that the present results are closely aligned with earlier findings. Considering the major uncertainties of the present experimental study (i.e., different pedestrians and substructures, smartphone-based registrations, limited number of gaits, etc.), this kind of outcome can confirm the validity and robustness of the original SMD proposal, as outlined in Section 2.

In particular, as shown in Figure 11a, the analysis of SMD results in terms of SDOF stiffness *k* confirmed a mostly linear variation of *k* with the walking frequency *f_p_* of each one of the involved pedestrians. Compared to p_1_ from [10], it can be perceived that a lower mass of pedestrian corresponds to a minimum reduction in the measured stiffness *k* for the biodynamic model (i.e., p_2_ walking on the concrete slab) at a given *f_p_*. When the experimental results for p_2_ pedestrian moving on the floor prototype are taken into account, a good correlation can still be observed with *k* trends for p_1_ moving on the rigid concrete slab. This finding suggests the accuracy of smartphone-based body CoM acquisitions in place of more accurate and refined sensors for similar applications (i.e., Table 1).

Finally, for the p_3_ volunteer walking on the timber floor, the measured stiffness *k*, as shown in Figure 11a, showed a further decrease compared to previous results for a given frequency. Notably, p_3_ walked slower than p_2_, and some variability in the SMD-calibrated parameters could also be attributed to individual motion features.

In terms of the calculated *ξ*-*c* trends for the experiments, the collected results are presented in Figure 11b. As in accordance with [10], it is possible to see that the trend is mostly linear, and there is a rather good match with previous p_1_ findings, both for p_2_ and p_3_ volunteers. From a practical point of view, according to Figure 11 and Equations (7)–(9), the empirical formulae for SMD modelling can be thus expressed as:

(11) $$k=A_{1}f_{p}-A_{2}$$

and

(12) $$\xi =B_{1}-B_{2}c$$

where the corresponding input parameters and coefficients of determination R^2^ are reported in Table 3.

It can be seen that when the cumulative experimental records are taken into account for fitting, the corresponding R^2^ for calibrating the stiffness *k* slightly modifies with the type of floor. Combining all the available experimental results for damping parameters is indeed more affected by variations of floor type.

It is important to note that the basic assumption for the present calibration approach, as shown in Equations (11) and (12) and in Section 2, is a linear fit for the empirical derivation of SMD parameters. This derives from the operational steps and governing equations of Section 2, considering that:

- the stiffness *k* of the pedestrian is first calculated from the experimental records of each gait, according to Equations (1) and (2), with *m* = *M*:

(13) $$k=\frac{F_{Z}\left(t\right)}{∆h(t)}=\frac{m\;a_{Z}\left(t\right)}{∆h(t)}$$

- the frequency *f_p_* is also derived from experimental records, and the *k*—*f*_p_ correlation, as shown in Equation (11), is defined;
- following the preliminary steps, the natural pulsation of the pedestrian, *ω_m_*, can be expressed as:

(14) $$\omega_{m}=\sqrt{\frac{k}{m}}=2\pi f_{m}$$

- where *k* and *m* are known, and:

(15) $$f_{m}=\frac{\omega_{m}}{2\pi }$$

- from iterative calculations, for a given pedestrian and walk, it can be found that there is only one value of the damping ratio *ξ* able to satisfy the system of governing equations of the problem, given that:

(16) $$c=2\;\xi \;m\;\omega_{m}$$

and

(17) $$\omega_{md}=\omega_{m}-\sqrt{1-2\xi^{2}}$$

at an assigned average frequency *f_p_* (Equation (10)).

Overall, considering the large number of influencing parameters for the examined problem, the obtained correlation in Table 3 looks rather satisfactory and poses the basis for further experimental investigations. From Table 3, it can be seen that R^2^ is generally close to the unit, while in a few cases the match of experimental data is less satisfactory. Most importantly, R^2^ decreases for *k* estimates as a function of *f_p_* for both p_2_ and p_3_ pedestrians on the timber floor (0.83 and 0.70, respectively), while it is still close to the unit for p_2_ on the rigid concrete slab (0.98). When the cumulative p_2_ + p_3_ records on the timber floor are taken into account (which means up to 100 post-processed gaits from 23 walks), the correlation increases up to 0.89 for stiffness predictions. This suggests the need for a sufficiently wide set of records, both in terms of the number of gaits and walking configurations and possibly pedestrians, for further verification. For general applications, the number of available records should also be distributed on a sufficiently extended range of walking frequencies *f_p_*.

**Figure 12:**
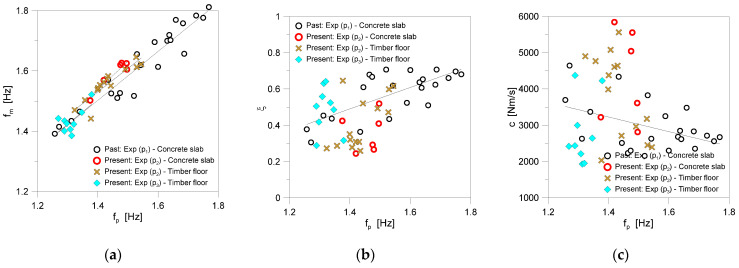
Trend of calculated parameters as a function of the walking frequency *f_p_*: (**a**) pedestrian frequency *f_m_*, (**b**) damping ratio *ξ*, and (**c**) damping coefficient *c*.

In support of this consideration, the trend of other relevant parameters is also shown in Figure 12, as a function of *f_p_*. The comparative dots, which represent the average of each walk for a given pedestrian and substructure, show the different distribution and interval *f_p_*. It can be seen that the damping terms are especially more scattered towards *f_p_*, which directly derives from a combination of influencing parameters.

Based on the present and past experimental evidence for the *ξ*—*f_p_* correlation, Table 4 shows a marked sensitivity and linear proportionality of the coefficient of determination R^2^ to the number of walks, where it is assumed that:

(18) $$\xi =C_{1}f_{p}-C_{2}$$

For preliminary estimates of the damping ratio *ξ*, the input coefficients given by the fitting of all the experimental results (p_1_ + p_2_ + p_3_) are herein suggested, where C_1_ = 0.5983 and C_2_ = 0.3865.

### 4.3. Effect on Structures

Regarding the general analysis of the experimental results in terms of the substructure effects, it is important to remember that both the mass and deformability of pedestrian systems are key influencing parameters for HSI phenomena [1,11]. The implicit limitations of the present experimental results may derive, in this sense, from the lack of multiple substructures/complex structural systems and/or a wide group of volunteers.

In terms of the estimated biodynamic features for normal walking frequencies (i.e., ≈1.402 Hz, the average *f_p_*), the comparative results in Figure 11 and Figure 12 and Table 3 are observed to be certainly affected by the structural background (i.e., the concrete slab or timber floor for p_2_). This can be noted, for example, in terms of higher damping predictions for the timber floor, rather than the concrete slab. The same results were observed to suffer slightly for the pedestrian mass (70 kg for both p_2_ and p_3_), especially compared to p_1_. Indeed, compared to [10], the estimated SMD parameters resulted in being mostly affected by the intrinsic motion features of each volunteer and by the number of post-processed gaits (due to floor span limitations and range of walking frequencies). Future studies will be consequently carried out in this direction.

**Figure 13:**
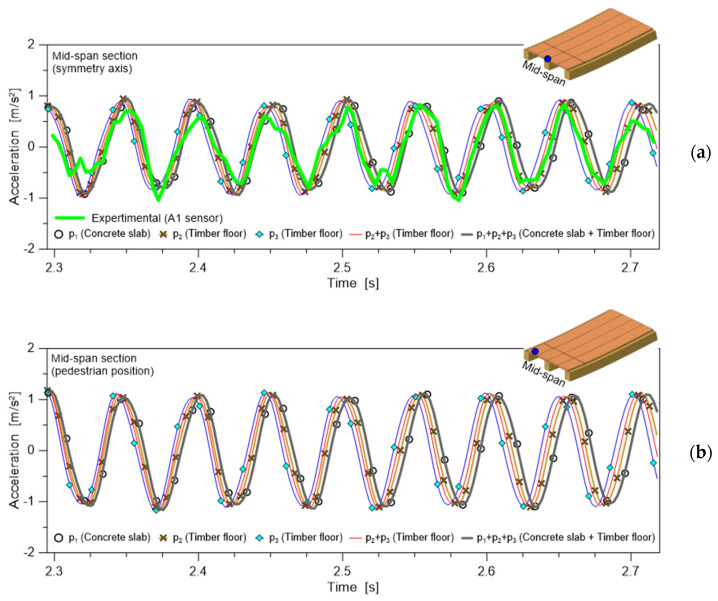
Example of numerically simulated human-induced effect on the timber floor prototype, according to the SMD modelling approach of Table 3 and Table 4: vertical acceleration at mid-span (**a**) on the symmetry section (A1 position) or (**b**) in the pedestrian position (left side of the floor).

From a practical point of view, it is however important at least to preliminarily point out the effects that can be expected from the input parameters discussed herein, in terms of structural response. In this regard, Figure 13 shows an example of numerical results that have been derived from time history analyses carried out in ABAQUS/Standard for the timber floor setup presented in Figure 5. The comparative structural analysis is carried out by applying human-induced vertical reaction forces with a SMD model according to Table 3 and Table 4 (with *f_p_*= 1.5 Hz, the assigned walking frequency and a pedestrian assumed to move linearly on the left side of the floor), while the structural response of the floor itself is verified in terms of the maximum vertical acceleration at the mid-span section.

In this regard, Figure 13a refers to the symmetry section, which coincides with the location of the A1 sensor for the experimental setup. Accordingly, the numerical predictions are compared with a selection of experimental measurements in A1 (i.e., Figure 8a). Similarly, Figure 13b shows the numerically predicted vertical accelerations on the left side of the floor, where the pedestrian was assumed to walk. Especially from Figure 13a, it is possible to see the potential of the present modelling strategy and calibration, as well as the sensitivity of numerical estimates to specific input parameters. Overall, these outcomes suggest the robustness of the presently discussed approach, as well as the definition of additional studies for a possible generalized extension of the procedure.

## 5. Conclusions and Future Work

For pedestrian systems, the analysis of Human-Structure Interaction (HSI) phenomena and human-induced reactions forces can be particularly challenging, and even require the use of sophisticated calculation and verification approaches. This issue is particularly relevant for pedestrian systems that are more sensitive to vibrations, such as lightweight systems and/or low-frequency systems.

This paper explored and verified the optimisation of calibration steps for a widely consolidated Spring-Mass-Damper (SMD) biodynamic pedestrian modelling strategy in the literature, as well as the possible application of smartphone-based acquisitions for the derivation of its basic input parameters. To this aim, the robustness and validity of smartphone-based data were verified with the support of different pedestrian volunteers, as well as different substructures (i.e., a rigid concrete slab or a timber floor prototype, respectively). A major advantage for the present study was derived from a previously validated SMD calibration strategy, which was developed to take advantage of single-body CoM sensor acquisitions. At the same time, the availability of a full-scale timber floor prototype was exploited to run an extended campaign of non-destructive dynamic tests with different pedestrians and walking features.

The post-processing of smartphone-based body CoM acquisitions was carried out on a total of 145 gaits and verified against the past elaboration of 240 gaits. The analysis of the experimental results showed that many influencing parameters can possibly affect the calibration of a rather simple but efficient pedestrian model, such as the SMD model. Furthermore, a rather close match was generally obtained with earlier findings in terms of the quantitative estimation of basic SMD features. A major influencing parameter was detected in the minimum number of gaits and walks that should be post-processed for a generalised application of the smartphone-based approach, especially for its application in pedestrian systems that can be particularly sensitive to human-induced vibrations.

Finally, a preliminary comparison of numerical estimates based on the herein-discussed SMD model calibration was also presented in terms of expected structural response. The comparative results, whilst limited in number and requiring further extensions, confirmed the high potential of the approach. In this sense, future experimental applications will be considered to account for different substructures and pedestrians.

## Figures and Tables

**Table 1:** Selected parameters for the present SMD calibration.

Parameter	Literature Study [10]	Present Study	
Pedestrian	p_1_	p_2_	p_3_
Gender	Female	Female	Male
Age	39	33	38
Height [m]	1.85	1.82	1.75
Weight [kg]	80	70	70
Facility for tests	University of Trieste	University of L’Aquila	University of L’Aquila
Walking frequency range [Hz]	1.2–2	1.3–1.6	1.25–1.4
Substructure	Rigid concrete slab	Rigid concrete slab + timber floor prototype	Timber floor prototype
Gaits	300	42 (concrete slab) + 98 (timber floor)	63
Walks	30	6 (concrete slab) + 14 (timber floor)	9
Gaits for each walk	10	7	7
Post-processed gaits for SMD	8 × 30 = 240	5 × 6 = 30 (concrete slab) + 5 × 14 = 70 (timber floor)	5 × 9 = 45
Body CoM sensor	Wi-Fi MEMS triaxial accelerometer (BeanDevice^®^ Wilow^®^)	S1 device (iPhone 14)	S1 device (iPhone 14)
Sampling rate [Hz]	200	100	100

**Table 2:** Walking frequency parameters for the present investigation.

Pedestrian	Substructure	Post-Processed Gaits for SMD	Average Frequency *f_p_* [Hz]	Standard Deviation
p_2_	Concrete slab	30	1.456	±0.049
p_2_	Timber floor	70	1.435	±0.067
p_3_	Timber floor	45	1.312	±0.033
Total		145	1.402	±0.082

**Table 3:** Input constants for Equations (11) and (12) and corresponding coefficients of determination R^2^ for the present smartphone-based SMD calibration.

Pedestrian	Substructure	Gaits	Walks	A_1_	A_2_	B_1_	B_2_	R^2^	
								*k–f_p_*	*ξ–c*
p_1_	Concrete slab	240	30	8190	4315.8	1.0705	0.0002	0.91	0.84
p_2_	Concrete slab	30	6	8432	5030.5	0.7173	0.00008	0.98	0.95
p_2_	Timber floor	70	14	6900	2949.3	0.8670	0.0001	0.83	0.97
p_3_	Timber floor	45	9	9272	6545.3	0.8583	0.0001	0.70	0.97
p_2_ + p_3_	Timber floor	115	23	9108	6193.4	0.8285	0.0001	0.89	0.94
p_1_ + p_2_ + p_3_	Both	385	59	9403	6397.8	0.9127	0.0001	0.92	0.84

**Table 4:** Input constants for Equation (18) and corresponding coefficients of determination R^2^ for the present smartphone-based SMD calibration.

Pedestrian	Substructure	Gaits	Walks	C_1_	C_2_	R^2^
						*ξ–f_p_*
p_1_	Concrete slab	240	30	0.5915	0.3375	0.52
p_2_	Concrete slab	30	6	0.326	0.1153	0.15
p_2_	Timber floor	70	14	1.2717	1.4148	0.70
p_3_	Timber floor	45	9	1.498	0.7714	0.22
p_2_ + p_3_	Timber floor	115	23	1.0129	1.0653	0.24
p_1_ + p_2_ + p_3_	Both	385	59	0.5983	0.3865	0.30

## Data Availability

Data will be shared upon reasonable request.
